# Supplementary material for: Time-series transcriptome analysis identified differentially expressed genes in broiler chicken infected with mixed Eimeria species
Source: Front Genet. 2022 Aug 8;13:886781. doi: 10.3389/fgene.2022.886781 (PMC9393255; doi:10.3389/fgene.2022.886781)
Supplement: Supplementary file 2 [file DataSheet1.ZIP › 7dpi_KEGG.Gsea.1625115961950/gsea_report_for_0_1625115961950.html]

Report for 0 1625115961950 [GSEA]

| GS  follow link to MSigDB | GS DETAILS | SIZE | ES | NES | NOM p-val | FDR q-val | FWER p-val | RANK AT MAX | LEADING EDGE || 1 | KEGG\_NATURAL\_KILLER\_CELL\_MEDIATED\_CYTOTOXICITY | Details ... | 67 | -0.55 | -1.88 | 0.000 | 0.018 | 0.021 | 3641 | tags=60%, list=30%, signal=85% |
| 2 | KEGG\_MTOR\_SIGNALING\_PATHWAY | Details ... | 45 | -0.52 | -1.71 | 0.001 | 0.124 | 0.253 | 2540 | tags=47%, list=21%, signal=59% |
| 3 | KEGG\_MELANOMA | Details ... | 48 | -0.52 | -1.70 | 0.000 | 0.089 | 0.269 | 2415 | tags=42%, list=20%, signal=52% |
| 4 | KEGG\_T\_CELL\_RECEPTOR\_SIGNALING\_PATHWAY | Details ... | 81 | -0.48 | -1.70 | 0.000 | 0.069 | 0.275 | 3691 | tags=54%, list=31%, signal=78% |
| 5 | KEGG\_PHOSPHATIDYLINOSITOL\_SIGNALING\_SYSTEM | Details ... | 65 | -0.49 | -1.69 | 0.001 | 0.064 | 0.311 | 3934 | tags=58%, list=33%, signal=86% |
| 6 | KEGG\_JAK\_STAT\_SIGNALING\_PATHWAY | Details ... | 90 | -0.47 | -1.67 | 0.000 | 0.072 | 0.398 | 1677 | tags=37%, list=14%, signal=42% |
| 7 | KEGG\_FOLATE\_BIOSYNTHESIS | Details ... | 9 | -0.73 | -1.67 | 0.005 | 0.062 | 0.398 | 1458 | tags=56%, list=12%, signal=63% |
| 8 | KEGG\_ADHERENS\_JUNCTION | Details ... | 63 | -0.48 | -1.65 | 0.001 | 0.067 | 0.465 | 2970 | tags=41%, list=25%, signal=55% |
| 9 | KEGG\_INOSITOL\_PHOSPHATE\_METABOLISM | Details ... | 49 | -0.49 | -1.63 | 0.003 | 0.079 | 0.561 | 3934 | tags=59%, list=33%, signal=88% |
| 10 | KEGG\_NON\_SMALL\_CELL\_LUNG\_CANCER | Details ... | 44 | -0.50 | -1.62 | 0.006 | 0.081 | 0.607 | 3905 | tags=59%, list=33%, signal=87% |
| 11 | KEGG\_SMALL\_CELL\_LUNG\_CANCER | Details ... | 71 | -0.47 | -1.61 | 0.003 | 0.079 | 0.635 | 3613 | tags=52%, list=30%, signal=74% |
| 12 | KEGG\_GLIOMA | Details ... | 51 | -0.49 | -1.60 | 0.006 | 0.081 | 0.676 | 5077 | tags=73%, list=42%, signal=125% |
| 13 | KEGG\_HEMATOPOIETIC\_CELL\_LINEAGE | Details ... | 47 | -0.49 | -1.59 | 0.005 | 0.083 | 0.717 | 3487 | tags=47%, list=29%, signal=66% |
| 14 | KEGG\_CYTOKINE\_CYTOKINE\_RECEPTOR\_INTERACTION | Details ... | 134 | -0.43 | -1.59 | 0.000 | 0.079 | 0.727 | 3344 | tags=45%, list=28%, signal=61% |
| 15 | KEGG\_COLORECTAL\_CANCER | Details ... | 51 | -0.47 | -1.57 | 0.008 | 0.097 | 0.821 | 2457 | tags=37%, list=20%, signal=47% |
| 16 | KEGG\_TYPE\_II\_DIABETES\_MELLITUS | Details ... | 36 | -0.50 | -1.55 | 0.015 | 0.103 | 0.855 | 1743 | tags=39%, list=15%, signal=45% |
| 17 | KEGG\_FOCAL\_ADHESION | Details ... | 158 | -0.41 | -1.53 | 0.001 | 0.119 | 0.906 | 3613 | tags=41%, list=30%, signal=58% |
| 18 | KEGG\_FC\_EPSILON\_RI\_SIGNALING\_PATHWAY | Details ... | 56 | -0.46 | -1.53 | 0.011 | 0.120 | 0.920 | 3613 | tags=50%, list=30%, signal=71% |
| 19 | KEGG\_PATHWAYS\_IN\_CANCER | Details ... | 249 | -0.40 | -1.52 | 0.000 | 0.121 | 0.933 | 3196 | tags=38%, list=27%, signal=51% |
| 20 | KEGG\_PANCREATIC\_CANCER | Details ... | 57 | -0.45 | -1.52 | 0.011 | 0.122 | 0.943 | 3613 | tags=51%, list=30%, signal=72% |
| 21 | KEGG\_ECM\_RECEPTOR\_INTERACTION |  | 64 | -0.45 | -1.52 | 0.010 | 0.117 | 0.945 | 3186 | tags=42%, list=27%, signal=57% |
| 22 | KEGG\_ENDOMETRIAL\_CANCER |  | 44 | -0.47 | -1.51 | 0.018 | 0.118 | 0.955 | 3905 | tags=55%, list=33%, signal=81% |
| 23 | KEGG\_PROGESTERONE\_MEDIATED\_OOCYTE\_MATURATION |  | 67 | -0.44 | -1.51 | 0.010 | 0.115 | 0.957 | 3759 | tags=46%, list=31%, signal=67% |
| 24 | KEGG\_REGULATION\_OF\_ACTIN\_CYTOSKELETON |  | 155 | -0.40 | -1.50 | 0.001 | 0.125 | 0.974 | 3186 | tags=37%, list=27%, signal=50% |
| 25 | KEGG\_HISTIDINE\_METABOLISM |  | 22 | -0.53 | -1.49 | 0.031 | 0.123 | 0.977 | 3000 | tags=55%, list=25%, signal=73% |
| 26 | KEGG\_ADIPOCYTOKINE\_SIGNALING\_PATHWAY |  | 52 | -0.45 | -1.49 | 0.018 | 0.127 | 0.984 | 1781 | tags=31%, list=15%, signal=36% |
| 27 | KEGG\_ACUTE\_MYELOID\_LEUKEMIA |  | 46 | -0.45 | -1.48 | 0.025 | 0.123 | 0.984 | 3613 | tags=54%, list=30%, signal=77% |
| 28 | KEGG\_PRIMARY\_IMMUNODEFICIENCY |  | 24 | -0.51 | -1.48 | 0.036 | 0.123 | 0.986 | 2408 | tags=42%, list=20%, signal=52% |
| 29 | KEGG\_FC\_GAMMA\_R\_MEDIATED\_PHAGOCYTOSIS |  | 74 | -0.42 | -1.46 | 0.015 | 0.137 | 0.993 | 3308 | tags=41%, list=28%, signal=56% |
| 30 | KEGG\_CHEMOKINE\_SIGNALING\_PATHWAY |  | 119 | -0.40 | -1.46 | 0.007 | 0.136 | 0.994 | 3621 | tags=47%, list=30%, signal=67% |
| 31 | KEGG\_OLFACTORY\_TRANSDUCTION |  | 14 | -0.57 | -1.45 | 0.054 | 0.141 | 0.996 | 4627 | tags=79%, list=39%, signal=128% |
| 32 | KEGG\_B\_CELL\_RECEPTOR\_SIGNALING\_PATHWAY |  | 57 | -0.43 | -1.44 | 0.028 | 0.153 | 0.998 | 3641 | tags=51%, list=30%, signal=73% |
| 33 | KEGG\_CHRONIC\_MYELOID\_LEUKEMIA |  | 61 | -0.42 | -1.43 | 0.028 | 0.158 | 0.998 | 2666 | tags=38%, list=22%, signal=48% |
| 34 | KEGG\_LONG\_TERM\_POTENTIATION |  | 47 | -0.43 | -1.41 | 0.043 | 0.189 | 1.000 | 4864 | tags=55%, list=40%, signal=93% |
| 35 | KEGG\_APOPTOSIS |  | 63 | -0.41 | -1.41 | 0.030 | 0.190 | 1.000 | 3613 | tags=48%, list=30%, signal=68% |
| 36 | KEGG\_NOD\_LIKE\_RECEPTOR\_SIGNALING\_PATHWAY |  | 37 | -0.45 | -1.40 | 0.052 | 0.190 | 1.000 | 2666 | tags=41%, list=22%, signal=52% |
| 37 | KEGG\_MAPK\_SIGNALING\_PATHWAY |  | 179 | -0.37 | -1.40 | 0.006 | 0.188 | 1.000 | 3655 | tags=39%, list=30%, signal=55% |
| 38 | KEGG\_CELL\_ADHESION\_MOLECULES\_CAMS |  | 73 | -0.40 | -1.40 | 0.034 | 0.191 | 1.000 | 2623 | tags=34%, list=22%, signal=44% |
| 39 | KEGG\_ENDOCYTOSIS |  | 141 | -0.38 | -1.39 | 0.012 | 0.187 | 1.000 | 4892 | tags=52%, list=41%, signal=86% |
| 40 | KEGG\_PRION\_DISEASES |  | 22 | -0.49 | -1.39 | 0.079 | 0.195 | 1.000 | 4135 | tags=55%, list=34%, signal=83% |
| 41 | KEGG\_PROSTATE\_CANCER |  | 75 | -0.39 | -1.38 | 0.040 | 0.198 | 1.000 | 3613 | tags=43%, list=30%, signal=61% |
| 42 | KEGG\_TRYPTOPHAN\_METABOLISM |  | 26 | -0.47 | -1.38 | 0.074 | 0.196 | 1.000 | 2698 | tags=42%, list=22%, signal=54% |
| 43 | KEGG\_TOLL\_LIKE\_RECEPTOR\_SIGNALING\_PATHWAY |  | 67 | -0.40 | -1.38 | 0.042 | 0.193 | 1.000 | 2666 | tags=39%, list=22%, signal=50% |
| 44 | KEGG\_GLYCEROLIPID\_METABOLISM |  | 36 | -0.44 | -1.38 | 0.064 | 0.190 | 1.000 | 3768 | tags=39%, list=31%, signal=56% |
| 45 | KEGG\_NEUROTROPHIN\_SIGNALING\_PATHWAY |  | 99 | -0.38 | -1.37 | 0.031 | 0.190 | 1.000 | 5180 | tags=55%, list=43%, signal=95% |
| 46 | KEGG\_MELANOGENESIS |  | 63 | -0.40 | -1.37 | 0.052 | 0.189 | 1.000 | 4993 | tags=54%, list=42%, signal=92% |
| 47 | KEGG\_GAP\_JUNCTION |  | 63 | -0.40 | -1.36 | 0.058 | 0.198 | 1.000 | 4806 | tags=44%, list=40%, signal=74% |
| 48 | KEGG\_INSULIN\_SIGNALING\_PATHWAY |  | 107 | -0.37 | -1.35 | 0.033 | 0.216 | 1.000 | 4008 | tags=46%, list=33%, signal=68% |
| 49 | KEGG\_COMPLEMENT\_AND\_COAGULATION\_CASCADES |  | 40 | -0.42 | -1.33 | 0.089 | 0.242 | 1.000 | 2237 | tags=33%, list=19%, signal=40% |
| 50 | KEGG\_ANTIGEN\_PROCESSING\_AND\_PRESENTATION |  | 26 | -0.45 | -1.33 | 0.109 | 0.239 | 1.000 | 2239 | tags=38%, list=19%, signal=47% |
| 51 | KEGG\_ARRHYTHMOGENIC\_RIGHT\_VENTRICULAR\_CARDIOMYOPATHY\_ARVC |  | 52 | -0.40 | -1.33 | 0.083 | 0.240 | 1.000 | 3918 | tags=37%, list=33%, signal=54% |
| 52 | KEGG\_DILATED\_CARDIOMYOPATHY |  | 54 | -0.40 | -1.32 | 0.088 | 0.253 | 1.000 | 3104 | tags=30%, list=26%, signal=40% |
| 53 | KEGG\_AXON\_GUIDANCE |  | 100 | -0.36 | -1.31 | 0.055 | 0.252 | 1.000 | 5127 | tags=54%, list=43%, signal=93% |
| 54 | KEGG\_BLADDER\_CANCER |  | 32 | -0.43 | -1.30 | 0.126 | 0.266 | 1.000 | 3196 | tags=44%, list=27%, signal=59% |
| 55 | KEGG\_OTHER\_GLYCAN\_DEGRADATION |  | 12 | -0.52 | -1.30 | 0.149 | 0.276 | 1.000 | 4074 | tags=50%, list=34%, signal=76% |
| 56 | KEGG\_VASCULAR\_SMOOTH\_MUSCLE\_CONTRACTION |  | 75 | -0.37 | -1.29 | 0.085 | 0.276 | 1.000 | 2416 | tags=27%, list=20%, signal=33% |
| 57 | KEGG\_GLYCOSAMINOGLYCAN\_BIOSYNTHESIS\_HEPARAN\_SULFATE |  | 18 | -0.47 | -1.27 | 0.153 | 0.306 | 1.000 | 5140 | tags=72%, list=43%, signal=126% |
| 58 | KEGG\_CALCIUM\_SIGNALING\_PATHWAY |  | 118 | -0.35 | -1.27 | 0.072 | 0.319 | 1.000 | 1461 | tags=21%, list=12%, signal=24% |
| 59 | KEGG\_GLYCOSPHINGOLIPID\_BIOSYNTHESIS\_GANGLIO\_SERIES |  | 13 | -0.50 | -1.26 | 0.184 | 0.318 | 1.000 | 3178 | tags=46%, list=26%, signal=63% |
| 60 | KEGG\_GNRH\_SIGNALING\_PATHWAY |  | 66 | -0.37 | -1.26 | 0.112 | 0.316 | 1.000 | 4905 | tags=59%, list=41%, signal=99% |
| 61 | KEGG\_LONG\_TERM\_DEPRESSION |  | 42 | -0.39 | -1.25 | 0.151 | 0.329 | 1.000 | 2241 | tags=33%, list=19%, signal=41% |
| 62 | KEGG\_ERBB\_SIGNALING\_PATHWAY |  | 69 | -0.36 | -1.24 | 0.130 | 0.354 | 1.000 | 1743 | tags=26%, list=15%, signal=30% |
| 63 | KEGG\_ALLOGRAFT\_REJECTION |  | 7 | -0.57 | -1.20 | 0.233 | 0.427 | 1.000 | 2345 | tags=43%, list=20%, signal=53% |
| 64 | KEGG\_STEROID\_HORMONE\_BIOSYNTHESIS |  | 16 | -0.45 | -1.20 | 0.230 | 0.424 | 1.000 | 2278 | tags=38%, list=19%, signal=46% |
| 65 | KEGG\_ABC\_TRANSPORTERS |  | 33 | -0.39 | -1.19 | 0.228 | 0.442 | 1.000 | 2842 | tags=42%, list=24%, signal=55% |
| 66 | KEGG\_NEUROACTIVE\_LIGAND\_RECEPTOR\_INTERACTION |  | 121 | -0.32 | -1.19 | 0.156 | 0.440 | 1.000 | 3734 | tags=36%, list=31%, signal=51% |
| 67 | KEGG\_AUTOIMMUNE\_THYROID\_DISEASE |  | 8 | -0.54 | -1.18 | 0.255 | 0.444 | 1.000 | 484 | tags=25%, list=4%, signal=26% |
| 68 | KEGG\_GRAFT\_VERSUS\_HOST\_DISEASE |  | 6 | -0.58 | -1.18 | 0.249 | 0.439 | 1.000 | 2345 | tags=50%, list=20%, signal=62% |
| 69 | KEGG\_RENAL\_CELL\_CARCINOMA |  | 62 | -0.35 | -1.18 | 0.212 | 0.444 | 1.000 | 5112 | tags=56%, list=43%, signal=98% |
| 70 | KEGG\_PPAR\_SIGNALING\_PATHWAY |  | 52 | -0.35 | -1.17 | 0.233 | 0.458 | 1.000 | 4053 | tags=46%, list=34%, signal=69% |
| 71 | KEGG\_NOTCH\_SIGNALING\_PATHWAY |  | 37 | -0.37 | -1.17 | 0.245 | 0.454 | 1.000 | 2592 | tags=30%, list=22%, signal=38% |
| 72 | KEGG\_HYPERTROPHIC\_CARDIOMYOPATHY\_HCM |  | 52 | -0.35 | -1.16 | 0.239 | 0.461 | 1.000 | 3400 | tags=27%, list=28%, signal=37% |
| 73 | KEGG\_LEUKOCYTE\_TRANSENDOTHELIAL\_MIGRATION |  | 81 | -0.33 | -1.16 | 0.227 | 0.471 | 1.000 | 4152 | tags=44%, list=35%, signal=67% |
| 74 | KEGG\_SYSTEMIC\_LUPUS\_ERYTHEMATOSUS |  | 22 | -0.40 | -1.15 | 0.281 | 0.477 | 1.000 | 2600 | tags=27%, list=22%, signal=35% |
| 75 | KEGG\_CELL\_CYCLE |  | 103 | -0.31 | -1.14 | 0.231 | 0.491 | 1.000 | 4419 | tags=45%, list=37%, signal=70% |
| 76 | KEGG\_NON\_HOMOLOGOUS\_END\_JOINING |  | 11 | -0.48 | -1.14 | 0.305 | 0.490 | 1.000 | 4024 | tags=64%, list=34%, signal=96% |
| 77 | KEGG\_GLYCOSPHINGOLIPID\_BIOSYNTHESIS\_GLOBO\_SERIES |  | 12 | -0.46 | -1.13 | 0.314 | 0.502 | 1.000 | 4067 | tags=58%, list=34%, signal=88% |
| 78 | KEGG\_OOCYTE\_MEIOSIS |  | 82 | -0.32 | -1.13 | 0.276 | 0.507 | 1.000 | 4627 | tags=41%, list=39%, signal=67% |
| 79 | KEGG\_VASOPRESSIN\_REGULATED\_WATER\_REABSORPTION |  | 37 | -0.36 | -1.12 | 0.310 | 0.514 | 1.000 | 5695 | tags=54%, list=47%, signal=102% |
| 80 | KEGG\_DORSO\_VENTRAL\_AXIS\_FORMATION |  | 16 | -0.42 | -1.12 | 0.324 | 0.510 | 1.000 | 1692 | tags=31%, list=14%, signal=36% |
| 81 | KEGG\_PRIMARY\_BILE\_ACID\_BIOSYNTHESIS |  | 11 | -0.47 | -1.12 | 0.329 | 0.503 | 1.000 | 5007 | tags=64%, list=42%, signal=109% |
| 82 | KEGG\_VEGF\_SIGNALING\_PATHWAY |  | 55 | -0.33 | -1.10 | 0.309 | 0.534 | 1.000 | 5026 | tags=60%, list=42%, signal=103% |
| 83 | KEGG\_P53\_SIGNALING\_PATHWAY |  | 55 | -0.33 | -1.10 | 0.322 | 0.532 | 1.000 | 1773 | tags=25%, list=15%, signal=30% |
| 84 | KEGG\_THYROID\_CANCER |  | 24 | -0.38 | -1.10 | 0.348 | 0.538 | 1.000 | 1692 | tags=25%, list=14%, signal=29% |
| 85 | KEGG\_REGULATION\_OF\_AUTOPHAGY |  | 17 | -0.41 | -1.10 | 0.350 | 0.537 | 1.000 | 4561 | tags=59%, list=38%, signal=95% |
| 86 | KEGG\_RIBOFLAVIN\_METABOLISM |  | 12 | -0.44 | -1.09 | 0.363 | 0.551 | 1.000 | 3244 | tags=50%, list=27%, signal=68% |
| 87 | KEGG\_LYSINE\_DEGRADATION |  | 35 | -0.35 | -1.08 | 0.361 | 0.553 | 1.000 | 3200 | tags=40%, list=27%, signal=54% |
| 88 | KEGG\_SPHINGOLIPID\_METABOLISM |  | 31 | -0.35 | -1.07 | 0.379 | 0.573 | 1.000 | 4467 | tags=58%, list=37%, signal=92% |
| 89 | KEGG\_WNT\_SIGNALING\_PATHWAY |  | 104 | -0.30 | -1.07 | 0.357 | 0.571 | 1.000 | 4455 | tags=38%, list=37%, signal=61% |
| 90 | KEGG\_GLYCEROPHOSPHOLIPID\_METABOLISM |  | 58 | -0.32 | -1.07 | 0.376 | 0.572 | 1.000 | 4467 | tags=41%, list=37%, signal=66% |
| 91 | KEGG\_LEISHMANIA\_INFECTION |  | 40 | -0.33 | -1.05 | 0.401 | 0.593 | 1.000 | 3166 | tags=38%, list=26%, signal=51% |
| 92 | KEGG\_UBIQUITIN\_MEDIATED\_PROTEOLYSIS |  | 111 | -0.29 | -1.05 | 0.396 | 0.600 | 1.000 | 4419 | tags=40%, list=37%, signal=62% |
| 93 | KEGG\_RENIN\_ANGIOTENSIN\_SYSTEM |  | 13 | -0.42 | -1.04 | 0.432 | 0.606 | 1.000 | 5757 | tags=77%, list=48%, signal=148% |
| 94 | KEGG\_VIRAL\_MYOCARDITIS |  | 33 | -0.33 | -1.03 | 0.445 | 0.629 | 1.000 | 2345 | tags=24%, list=20%, signal=30% |
| 95 | KEGG\_O\_GLYCAN\_BIOSYNTHESIS |  | 22 | -0.36 | -1.03 | 0.447 | 0.625 | 1.000 | 2617 | tags=36%, list=22%, signal=46% |
| 96 | KEGG\_TGF\_BETA\_SIGNALING\_PATHWAY |  | 62 | -0.30 | -1.02 | 0.442 | 0.628 | 1.000 | 3306 | tags=37%, list=28%, signal=51% |
| 97 | KEGG\_PROXIMAL\_TUBULE\_BICARBONATE\_RECLAMATION |  | 16 | -0.38 | -1.02 | 0.451 | 0.631 | 1.000 | 520 | tags=19%, list=4%, signal=20% |
| 98 | KEGG\_TIGHT\_JUNCTION |  | 89 | -0.28 | -1.01 | 0.473 | 0.651 | 1.000 | 3948 | tags=37%, list=33%, signal=55% |
| 99 | KEGG\_ALDOSTERONE\_REGULATED\_SODIUM\_REABSORPTION |  | 28 | -0.34 | -1.01 | 0.471 | 0.647 | 1.000 | 2754 | tags=46%, list=23%, signal=60% |
| 100 | KEGG\_NUCLEOTIDE\_EXCISION\_REPAIR |  | 38 | -0.32 | -1.00 | 0.479 | 0.653 | 1.000 | 5370 | tags=58%, list=45%, signal=104% |
| 101 | KEGG\_GLYCOSAMINOGLYCAN\_BIOSYNTHESIS\_CHONDROITIN\_SULFATE |  | 17 | -0.37 | -0.99 | 0.493 | 0.661 | 1.000 | 3406 | tags=35%, list=28%, signal=49% |
| 102 | KEGG\_BASAL\_CELL\_CARCINOMA |  | 35 | -0.32 | -0.99 | 0.496 | 0.655 | 1.000 | 2811 | tags=26%, list=23%, signal=33% |
| 103 | KEGG\_CIRCADIAN\_RHYTHM\_MAMMAL |  | 12 | -0.40 | -0.99 | 0.496 | 0.664 | 1.000 | 4432 | tags=50%, list=37%, signal=79% |
| 104 | KEGG\_N\_GLYCAN\_BIOSYNTHESIS |  | 40 | -0.31 | -0.97 | 0.531 | 0.695 | 1.000 | 1497 | tags=18%, list=12%, signal=20% |
| 105 | KEGG\_HOMOLOGOUS\_RECOMBINATION |  | 23 | -0.33 | -0.95 | 0.536 | 0.716 | 1.000 | 5351 | tags=61%, list=45%, signal=110% |
| 106 | KEGG\_TYPE\_I\_DIABETES\_MELLITUS |  | 11 | -0.39 | -0.94 | 0.554 | 0.736 | 1.000 | 484 | tags=18%, list=4%, signal=19% |
| 107 | KEGG\_TAURINE\_AND\_HYPOTAURINE\_METABOLISM |  | 5 | -0.48 | -0.93 | 0.588 | 0.753 | 1.000 | 5060 | tags=60%, list=42%, signal=104% |
| 108 | KEGG\_NICOTINATE\_AND\_NICOTINAMIDE\_METABOLISM |  | 18 | -0.33 | -0.91 | 0.599 | 0.792 | 1.000 | 3943 | tags=44%, list=33%, signal=66% |
| 109 | KEGG\_FRUCTOSE\_AND\_MANNOSE\_METABOLISM |  | 28 | -0.30 | -0.89 | 0.638 | 0.813 | 1.000 | 4133 | tags=46%, list=34%, signal=71% |
| 110 | KEGG\_STARCH\_AND\_SUCROSE\_METABOLISM |  | 26 | -0.30 | -0.89 | 0.653 | 0.818 | 1.000 | 4203 | tags=54%, list=35%, signal=83% |
| 111 | KEGG\_ONE\_CARBON\_POOL\_BY\_FOLATE |  | 15 | -0.34 | -0.88 | 0.645 | 0.823 | 1.000 | 4242 | tags=40%, list=35%, signal=62% |
| 112 | KEGG\_RIG\_I\_LIKE\_RECEPTOR\_SIGNALING\_PATHWAY |  | 39 | -0.28 | -0.88 | 0.668 | 0.817 | 1.000 | 3547 | tags=41%, list=30%, signal=58% |
| 113 | KEGG\_PURINE\_METABOLISM |  | 112 | -0.24 | -0.88 | 0.726 | 0.810 | 1.000 | 3370 | tags=28%, list=28%, signal=38% |
| 114 | KEGG\_ASCORBATE\_AND\_ALDARATE\_METABOLISM |  | 7 | -0.39 | -0.84 | 0.692 | 0.867 | 1.000 | 986 | tags=29%, list=8%, signal=31% |
| 115 | KEGG\_GLYCOSYLPHOSPHATIDYLINOSITOL\_GPI\_ANCHOR\_BIOSYNTHESIS |  | 19 | -0.30 | -0.82 | 0.724 | 0.889 | 1.000 | 5828 | tags=53%, list=49%, signal=102% |
| 116 | KEGG\_DNA\_REPLICATION |  | 29 | -0.26 | -0.79 | 0.788 | 0.927 | 1.000 | 5351 | tags=48%, list=45%, signal=87% |
| 117 | KEGG\_PENTOSE\_PHOSPHATE\_PATHWAY |  | 19 | -0.28 | -0.78 | 0.780 | 0.934 | 1.000 | 4203 | tags=53%, list=35%, signal=81% |
| 118 | KEGG\_STEROID\_BIOSYNTHESIS |  | 12 | -0.30 | -0.74 | 0.805 | 0.977 | 1.000 | 68 | tags=8%, list=1%, signal=8% |
| 119 | KEGG\_INTESTINAL\_IMMUNE\_NETWORK\_FOR\_IGA\_PRODUCTION |  | 20 | -0.26 | -0.73 | 0.837 | 0.982 | 1.000 | 4158 | tags=30%, list=35%, signal=46% |
| 120 | KEGG\_BASAL\_TRANSCRIPTION\_FACTORS |  | 26 | -0.23 | -0.69 | 0.889 | 1.000 | 1.000 | 3523 | tags=23%, list=29%, signal=33% |
| 121 | KEGG\_HEDGEHOG\_SIGNALING\_PATHWAY |  | 33 | -0.22 | -0.68 | 0.909 | 1.000 | 1.000 | 4271 | tags=33%, list=36%, signal=52% |
| 122 | KEGG\_BETA\_ALANINE\_METABOLISM |  | 16 | -0.26 | -0.68 | 0.889 | 1.000 | 1.000 | 2534 | tags=31%, list=21%, signal=40% |
| 123 | KEGG\_SULFUR\_METABOLISM |  | 6 | -0.33 | -0.66 | 0.887 | 1.000 | 1.000 | 270 | tags=17%, list=2%, signal=17% |
| 124 | KEGG\_SNARE\_INTERACTIONS\_IN\_VESICULAR\_TRANSPORT |  | 30 | -0.22 | -0.65 | 0.930 | 1.000 | 1.000 | 3698 | tags=30%, list=31%, signal=43% |
| 125 | KEGG\_PEROXISOME |  | 70 | -0.19 | -0.65 | 0.964 | 1.000 | 1.000 | 4383 | tags=34%, list=36%, signal=54% |
| 126 | KEGG\_RETINOL\_METABOLISM |  | 23 | -0.22 | -0.64 | 0.924 | 1.000 | 1.000 | 2278 | tags=22%, list=19%, signal=27% |
| 127 | KEGG\_AMYOTROPHIC\_LATERAL\_SCLEROSIS\_ALS |  | 36 | -0.20 | -0.62 | 0.949 | 1.000 | 1.000 | 1804 | tags=17%, list=15%, signal=20% |
| 128 | KEGG\_RNA\_DEGRADATION |  | 49 | -0.18 | -0.60 | 0.983 | 1.000 | 1.000 | 2441 | tags=16%, list=20%, signal=20% |
| 129 | KEGG\_BUTANOATE\_METABOLISM |  | 21 | -0.21 | -0.58 | 0.961 | 1.000 | 1.000 | 3800 | tags=33%, list=32%, signal=49% |
| 130 | KEGG\_BIOSYNTHESIS\_OF\_UNSATURATED\_FATTY\_ACIDS |  | 19 | -0.21 | -0.58 | 0.958 | 1.000 | 1.000 | 4784 | tags=42%, list=40%, signal=70% |
| 131 | KEGG\_MISMATCH\_REPAIR |  | 20 | -0.21 | -0.57 | 0.968 | 1.000 | 1.000 | 6931 | tags=75%, list=58%, signal=177% |
| 132 | KEGG\_ASTHMA |  | 3 | -0.30 | -0.50 | 0.993 | 1.000 | 1.000 | 2836 | tags=33%, list=24%, signal=44% |
| 133 | KEGG\_BASE\_EXCISION\_REPAIR |  | 25 | -0.17 | -0.50 | 0.991 | 1.000 | 1.000 | 3376 | tags=20%, list=28%, signal=28% |
| 134 | KEGG\_PATHOGENIC\_ESCHERICHIA\_COLI\_INFECTION |  | 44 | -0.15 | -0.48 | 0.999 | 1.000 | 1.000 | 3691 | tags=20%, list=31%, signal=29% |
| 135 | KEGG\_SPLICEOSOME |  | 96 | -0.12 | -0.43 | 1.000 | 0.999 | 1.000 | 6242 | tags=40%, list=52%, signal=82% |
Table: Gene sets enriched in phenotype **0 (3 samples) **[plain text format]****
